# A new, deep quinoxaline-based cavitand receptor for the complexation of benzene

**DOI:** 10.1107/S2056989018017784

**Published:** 2019-01-04

**Authors:** Roberta Pinalli, Jakub W. Trzciński, Enrico Dalcanale, Chiara Massera

**Affiliations:** a Dipartimento di Scienze Chimiche, della Vita e della Sostenibilità Ambientale, Università di Parma, Parco Area delle Scienze 17/A, 43124 Parma, Italy

**Keywords:** crystal structure, quinoxaline cavitands, inclusion compounds, benzene

## Abstract

We report the synthesis of a new macrocyclic receptor, namely 2,8,14,20-tetra­hexyl-4,24:6,10:12,16:18,22-*O*,*O*′-tetra­kis­[2,3-di­hydro-[1,4]dioxino[2,3-*g*]quinoxalin-7,8-di­yl]resorcin[4]arene, **DeepQxCav**, obtained by the addition of ethyl­ene glycol di­tosyl­ate to an octa­hydroxy quinoxaline cavitand. A 1:1 supra­molecular complex of this cavitand with benzene has been obtained and analysed through X-ray diffraction analysis. The complex, of general formula C_92_H_88_O_16_N_8_·C_6_H_6_, crystallizes in the space group *C*2/*c*, with the cavitand host located about a twofold rotation axis. The benzene guest, which is held inside the cavity by C—H⋯π inter­actions and dispersion forces, is disordered over two equivalent sites, one in a general position and one lying on a twofold axis. The crystal structure features C—H⋯O hydrogen bonds and C—H⋯π inter­actions involving the alkyl chains, the aromatic rings, and the O atoms of the dioxane moiety of the resorcinarene scaffold. The crystal studied was refined as a two-component twin.

## Abstract 2

A new, deep tetra­quinoxaline-based cavitand has been synthesized, and its solid-state complex with benzene has been studied through X-ray diffraction analysis.

## Chemical context   

Resorcinarene-based cavitands are macrocyclic synthetic compounds (Cram, 1983 ▸; Cram & Cram, 1994 ▸), whose versatility primarily stems from the possibility of modifying the size and form of the cavity by choosing different bridging groups connecting the phenolic hydroxyl groups of the resorcinarene scaffold. This allows the tuning of the complexation properties of the cavity, which can thus inter­act with neutral and charged mol­ecules through hydrogen bonding, π–π stacking and C—H⋯π inter­actions, but also forms coordinate bonds with metal centers to create discrete complexes, cages or extended networks. These properties have made cavitands useful receptors for mol­ecular recognition and building blocks for crystal engineering (Pinalli *et al.*, 2016 ▸; Kane *et al.*, 2015 ▸; Brekalo *et al.*, 2018 ▸). In our group, we have been exploiting two main types of receptors, in which the bridging groups at the upper rim are either phospho­nate *R*PO_3_ moieties or quinoxaline ring systems. Both families have been extensively used in sensing in solution (Lee *et al.*, 2018 ▸; Liu *et al.* 2018 ▸) and in the gas phase (Melegari *et al.*, 2013 ▸; Tudisco *et al.*, 2016 ▸). Indeed, the demand for fast and reliable detection of bio­logical and chemical haza­rds is rising continuously and optimal sensors for environmental, security and biomedical applications must be sufficiently responsive to allow detection of the target analyte at low concentrations, and selective enough to respond primarily to a single chemical species in the presence of inter­ferents. In this respect, quinoxaline-based cavitands, exploiting the π-basicity and hydro­phobicity of their cavity are ideal hosts to inter­act with aromatic compounds (Pinalli *et al.*, 2018 ▸). Following this line of research, we have synthesized a new member of the quinoxaline family, **DeepQxCav**, in which the cavity has been made deeper by the addition of four 1,4 dioxane rings on the quinoxaline walls. In this paper we report and analyse the crystal structure of its complex with benzene as guest.
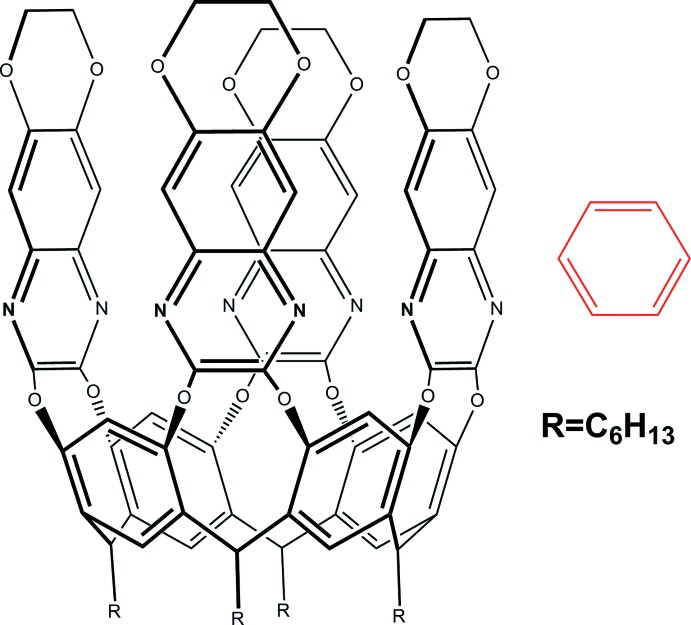


## Structural commentary   

**Figure 1:**
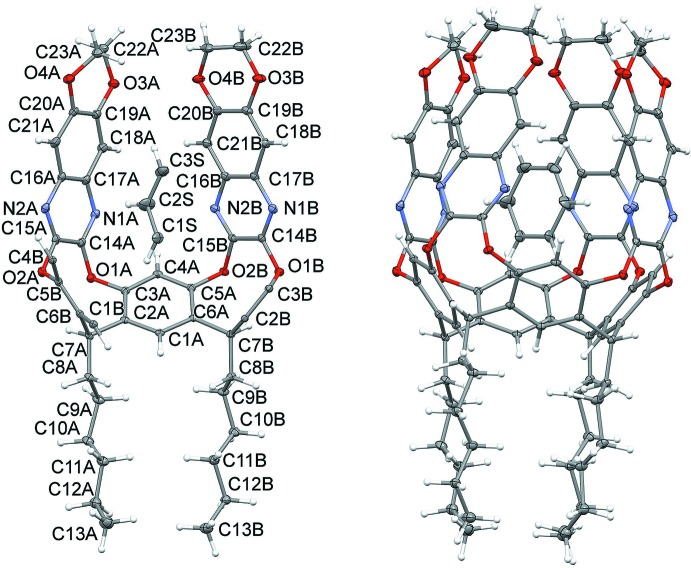
Left: asymmetric unit of the title compound with labelling scheme and ellipsoids drawn at the 20% probability level. Right: mol­ecular structure of the whole complex. The symmetry-related atoms are in position 1 − *x*, *y*, 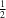 − *z*. For both views, only one of the two disordered orientations has been shown for clarity.

The mol­ecular structure of **DeepQxCav** is shown in Fig. 1 ▸. It consists of a 1:1 host–guest complex in which one mol­ecule of benzene is engulfed inside the walls of the cavitand. The complex crystallizes in the monoclinic crystalline system, in space group *C*2/*c*. The asymmetric unit comprises half of a cavitand host in a general position, about a twofold rotation axis, and half a mol­ecule of the benzene guest disordered over two sites, one in a general position and one lying on a twofold axis. Distances and angles are in good agreement with similar compounds reported in the literature (see *Section 4*).

**Figure 2:**
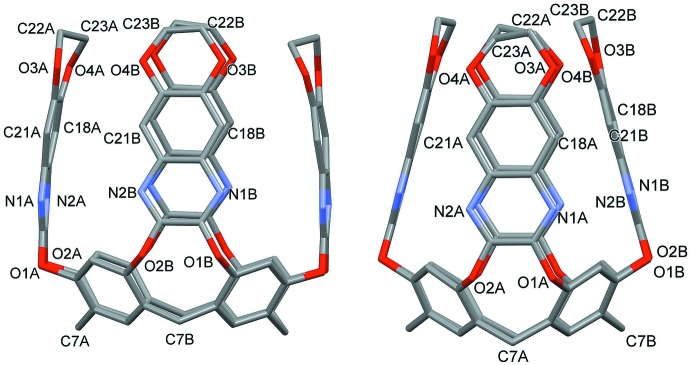
Perspective views of the cavity of **DeepQxCav** with partial labelling scheme, referred only to atoms in general positions. H atoms and alkyl chains have been omitted for clarity.

Fig. 2 ▸ shows two perspective views of the shape of the cavity (in a *vase* conformation) upon complexation of the guest. The depth of the cavity has been calculated as the distance between the mean plane passing through the groups of atoms C7 at the lower rim and C22—C23 of the upper rim, yielding a value of 10.290 (2) Å. The mean planes passing through the quinoxaline moieties are inclined with respect to the plane passing through the O1/O2 atoms (see Fig. 2 ▸), forming angles of 85.24 (3) and 75.16 (4)° for the walls labelled *A* and *B*, respectively. The mouth of the cavity is roughly rectangular in shape, but because of the bending of the walls, the opening is blocked by the steric hindrance of the four 1,4 dioxane rings (see Table 1 ▸ for geometrical details).

## Supra­molecular features   

**Figure 3:**
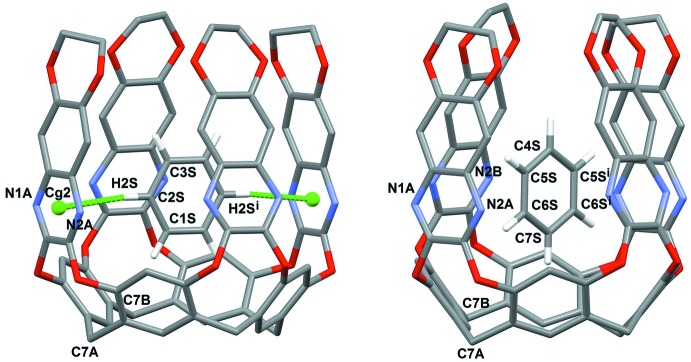
View of the inter­actions (green dotted lines) involving the benzene ring and the quinoxaline cavitand (both orientations of the guest are shown). Symmetry code: (i) 1 − *x*, *y*, 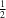 − *z*.

The most inter­esting supra­molecular feature of the title compound is the encapsulation of benzene inside the aromatic cavity of the host. As can be seen in Fig. 3 ▸, two orientations of the guest are present, from now on called I for ring C1*S*–C3*S* and II for ring C4*S*–C7*S* (the remaining atoms are generated by symmetry). I and II are rotated by *ca* 60° with respect to each other. In both cases, the benzene mol­ecule is found deep inside the cavity, at the same level of the pyrazine rings, roughly parallel to the walls labelled *B* [the angles formed by the mean planes passing through the benzene rings and through the quinoxaline wall are 82.4 (5) and 84.2 (3)° for I and II, respectively] and perpendicular to the walls labelled *A* [angles of 15.3 (2) and 15.0 (2)° for I and II, respectively]. In particular, the distances of C1*S* and C7*S* from the mean plane passing through the resorcinarene oxygen atoms O1/O2 are 1.128 (5) and 1.003 (9) Å, respectively. In the case of orientation I, two symmetry-related, equivalent C—H⋯π inter­actions are present, between C2*S*—H2*S* and the centroid C*g*2 of the ring (see Table 2 ▸). These inter­actions are absent in the case of orientation II, which is stabilized by van der Waals dispersion forces only.

**Figure 4:**
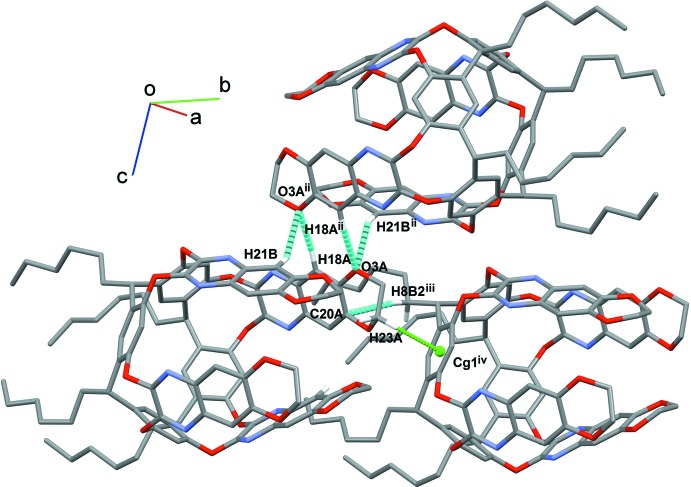
View of the relevant C—H⋯O hydrogen bonds and C—H⋯π inter­actions (light-blue and green dotted lines, respectively) stabilizing the crystal structure of the title compound. Only the H atoms involved in the inter­actions are shown. The benzene guest has been omitted for clarity.

In the crystal, the main inter­actions connecting the cavitands are C—H⋯O hydrogen bonds, involving the C—H groups of the alkyl chains (C12*B*—H12*D*) and of the aromatic rings (C18*A*—H18*A*, C21*B*—H21*B*) with the oxygen atoms of the dioxane moiety (O3*A*) or of the resorcinarene scaffold (O1*A*, see Figs. 4 ▸ and 5 ▸ and Table 2 ▸). Further consolidation of the structure is provided by C—H⋯π inter­actions (Fig. 4 ▸) due to the presence of aromatic rings in the cavitand scaffold.

## Database survey   

Several structures of quinoxaline-based cavitands have been published in recent years. A search in the Cambridge Structural Database (Version 5.38, update August 2018; Groom *et al.*, 2016 ▸) yielded 20 hits, of which 15 were inclusion compounds. In particular, the group of Professor Dalcanale has reported compounds NUTBUB01 and GURLIQ (Bertani *et al.*, 2016 ▸) in which a singly or doubly ‘roofed’ quinoxaline cavitand forms a 1:1 complex with benzene; LIMFOE and LIMGAR (Pinalli *et al.*, 2013 ▸) in which the guests are 1,3-benzodioxole and 5-allyl-1,3-benzodioxole, respectively; and the fluoro­benzene complex YAGVIL (Soncini *et al.*, 1992 ▸). Other related compounds are BUJNUR (Ballistreri *et al.*, 2016 ▸), a benzene clathrate co-crystallizing with fullerene; LUDJEA (Wagner *et al.*, 2009 ▸), in which the guest is phenyl azide; and UNIFAY (Azov *et al.*, 2003 ▸), an inclusion compound with aceto­nitrile, the only non-aromatic guest of the series.

**Figure 6:**
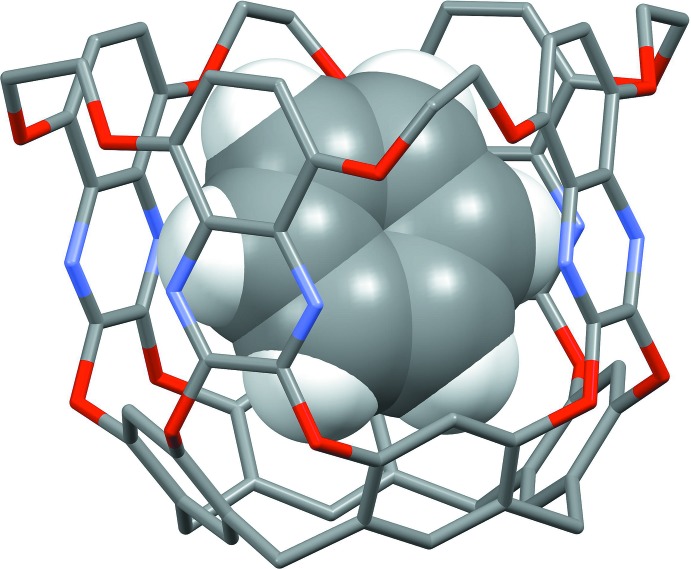
View of the orientation of benzene (in space-filling mode) inside the rigidified cavitand EtQxBox. Alkyl chains and host H atoms have been omitted for clarity.

Particularly inter­esting is a quinoxaline-based cavitand (EtQxBox) in which four ethyl­endi­oxy bridges between the quinoxaline wings have been introduced to obtain a rigidification of the cavity (Trzciński *et al.*, 2017 ▸). Also in that case, the crystal structure of the inclusion compound with benzene has been obtained and analysed in detail in the solid state. Differently from what happens in the title compound, the benzene molecule does not lie parallel to the quinoxaline walls of EtQxBox (Fig. 6 ▸) and is held inside the cavity by two C—H⋯π inter­actions with the lower aromatic part of the cavitand, and two bifurcated C—H⋯ N inter­actions with the nitro­gen atoms of two adjacent quinoxaline moieties. The shortest distance of a carbon atom of the guest from the mean plane passing through the O1/O2 groups of atoms is 1.268 (8) Å.

## Synthesis and crystallization   

All commercial reagents were ACS reagent grade and used as received. Solvents were dried and distilled using standard procedures. ^1^H NMR spectra were recorded on Bruker Avance 300 (300 MHz) and on Bruker Avance 400 (400 MHz) spectrometers. All chemical shifts (*δ*) were reported in parts per million (ppm) relative to proton resonances resulting from incomplete deuteration of NMR solvents. The Matrix-assisted laser desorption/ionization analyses (MALDI TOF–TOF) were performed on an AB SCIEX MALDI TOF–TOF 4800 Plus using α-cyano-4-hy­droxy­cinnamic acid as a matrix. The GC–Mass analyses were performed on a Hewlett–Packard Agilent 6890 series equipped in Supelco® SLBTM 5ms column and Hewlett–Packard 5973 MS Selective Mass Detector.

**Figure 7:**
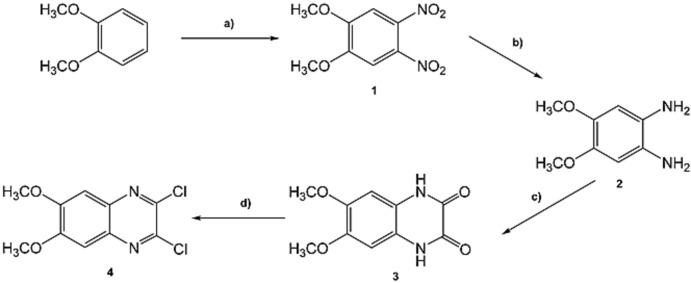
Synthesis of **4**: a) HNO_3_ 65%, 373 K, 8 h, 80%; b) Pd/C 10%, H_2_ 3 bar, EtOH, RT, 24 h, 100%; c) Oxalic acid, HCl 4 N, 373 K, 16 h, 77%; d) POCl_3_, di­chloro­ethane, 363 K, 16 h, 80%.

**Figure 8:**
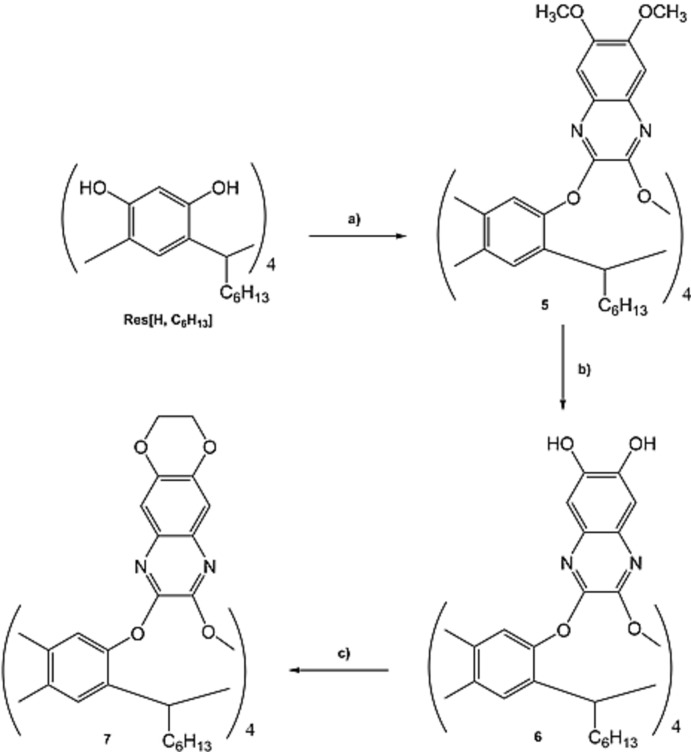
Synthesis of **7**: a) **4**, K_2_CO_3_, DMF, 393 K under microwave irradiation (300 W), 2 h, 92%; b) BBr_3_, dry chloro­form, 353 K, 24 h, 100%; c) ethyl­ene glycol di­tosyl­ate, Cs_2_CO_3_, DMF, 393 K under microwave irradiation (300 W), 1.5 h, 90%.

Cavitand QxCav (**7**) was prepared according to the following convergent synthetic approach: (i) synthesis of the 2,3-di­chloro-6,7-dimeth­oxy quinoxaline bridging unit **4** (Fig. 7 ▸); (ii) introduction of the dimeth­oxy-functionalized quinoxaline bridging unit onto the resorcinarene skeleton, deprotection of the meth­oxy groups and subsequent ring closure (Fig. 8 ▸).

The multistep synthesis of **4** started with nitration of veratrole following an electrophilic aromatic substitution reaction in concentrated nitric acid, under reflux. The obtained 1,2-dimeth­oxy-4,5-di­nitro benzene (**1**) was successively reduced using a catalytic amount of metallic Pd on activated carbon in an H_2_ atmosphere to give 1,2-dimeth­oxy-4,5-di­amino benzene (**2**). Due to the high reactivity of amino groups, compound **2** was used without any further purification for a condensation with oxalic acid under acidic conditions to give heterocycle **3**. The final step was the chlorination of the 6,7-di­meth­oxy­quinoxaline-2,3-dione (**3**) in the presence of POCl_3_ as chlorin­ating agent, di­methyl­formamide as catalyst and di­chloro­ethane as solvent. The functionalized bridging unit **4** was obtained in 80% yield after column chromatography.

As regards the resorcinarene scaffold (Res[H, C_6_H_13_]) for the preparation of the cavitand receptor, the one with hexyl feet was chosen as a compromise between solubility, which helps in the purification of inter­mediates and final products, and ease of crystallization. The synthesis consists of three steps (Fig. 8 ▸): firstly the hexyl-footed resorcinarene **5** (Tunstad *et al.*, 1989 ▸) was fourfold bridged with the 2,3-di­chloro-5,8-dimeth­oxy quinoxaline (**4**) under microwave irradiation, leading to octa­meth­oxy­quinoxaline cavitand (**5**) in 92% yield. The ^1^H NMR studies showed the fluctional *vase*–*kite* conformation of the cavitand **5**, due to the presence of the meth­oxy groups in the 6,7 positions relative to the quinoxaline moiety. The purified cavitand **6** was successively reacted with a Lewis acid (BBr_3_) in dry chloro­form under reflux, to cleave the methyl protecting groups of the quinoxaline walls. The deprotection of eight CH_3_ groups influences the cavitand conformation, as observed by the ^1^H NMR analysis, and the octa­hydroxy cavitand **6** is in the pure *vase* conformation. This change is due to the presence of hydrogen bonding between the hydroxyl groups placed at the cavity entrance. This strong inter­action tightens the cavity, holding it in the *vase* form. The last reaction step was the closure of the 1,4 dioxane ring by reacting the octa­hydroxy cavitand **6** and ethyl­ene glycol di­tosyl­ate under microwave irradiation in the presence of Cs_2_CO_3_ as base and di­methyl­formamide as solvent. Both ^1^H NMR and MALDI TOF–TOF analyses confirmed the formation of the desired compound.

**1,2-Dimeth­oxy-4,5-di­nitro benzene (1):** 1,2-Dimeth­oxy benzene (40 mmol) was added dropwise into a flask containing an aqueous solution of HNO_3_ 65% (25 mL) and stirred for 1 h at RT. A yellow precipitate was formed and the reaction was stirred at 373 K for an additional 8 h. The reaction was cooled to RT and the yellow emulsion was poured into a beaker containing ice-cooled water, filtered and dried under vacuum. The pure product **1** was obtained by a threefold recrystal­lization from glacial acetic acid in 80% yield.^1^H NMR (400 MHz, CDCl_3_): *δ* = 4.05 (*s*, 6H, **CH_3_**OAr), 7.35 (*s*, 2H, Ar**H**). GC–MS: *m*/*z* 229 [*M*]^+^.

**1,2-Dimeth­oxy-4,5-di­amino benzene (2):** To a suspension of compound **1** (30 mmol) in absolute ethanol (50 mL) a catalytic amount of palladium on charcoal (10%, *w*/*w*) was added. The reactor was mounted in a PARR hydrogenation apparatus and air atmosphere was replaced with H_2_ at 3 bar. The reaction was stirred at RT for 24 h. The product was filtered through celite, washed with ethanol and the solvent was removed under reduced pressure obtaining the final product **2** in quan­ti­tative yield. ^1^H NMR (400 MHz, CDCl_3_): *δ* (ppm) = 3.25 (*bs*, 4H, **H_2_**NAr), 3.80 (*s*, 6H, **CH_3_**OAr), 6.40 (*s*, 2H, Ar**H**). GC–MS: *m*/*z* = 169 [*M*]^+^.

**6,7-Di­meth­oxy­quinoxaline-2,3-dione (3)**: A solution of compound **2** in 4 *N* HCl (26 mmol, 1 eq.) was added to a stirring solution of oxalic acid (34 mmol, 1,3 eq.) in a 4 *N* HCl solution (33 mL) and refluxed for 16 h. After cooling to RT, the formed precipitate was filtered and dried under vacuum, giving the desired product **3** in 77% yield. ^1^H-NMR (300 MHz, DMSO-*d*_6_): *δ* (ppm) = 3.65 (*s*, 6H, **CH_3_**OAr), 6.72 (*s*, 2H, Ar**H**), 11.70 (*s*, 2H, CN**H**C). GC–MS: *m*/*z* = 223 [*M*]^+^.

**2,3-Dicloro-6,7-di­meth­oxy­quinoxaline (4):** 6,7-Di­meth­oxy­quinoxaline-2,3-dione **3** (20 mmol, 1 eq.), POCl_3_ (400 mmol, 20 eq.) and three drops of dry DMF were added into di­chloro­ethane (100 mL) and stirred at 363 K for 16 h. Subsequently, the solvent was removed under vacuum and the obtained solid was dissolved in di­chloro­methane and filtered through celite. The crude product was purified by flash chroma­tography giving the pure compound **4** in 80% yield. ^1^H-NMR (400 MHz, CDCl_3_): *δ* = 4.04 (*s*, 6H, **CH_3_**OAr), 7.25 (*s*, 2H, Ar**H**). GC–MS: *m*/*z* = 260 [M]^+^.

**Octa­meth­oxy quinoxaline cavitand (5):** Resorcinarene **Res[H, C_6_H_13_]** (0.35 mmol, 1eq.), 2,3-di­chloro-6,7-di­meth­oxy­quinoxaline **4** (0.51 mmol, 4,5 eq.), dry K_2_CO_3_ (1.87 mmol, 16 eq.) and dry DMF were added into an oven-dried microwave vessel under an Ar atmosphere and reacted under microwave irradiation at 393 K for 2 h. Afterwards, the mixture was extracted with di­chloro­methane/H_2_O and the organic fractions were collected, dried over Na_2_SO_4_ and the solvent was removed under reduced pressure. The crude product was purified by flash chromatography affording cavitand **5** in 92% yield.^1^H-NMR (300 MHz, CD_2_Cl_2_) – fluxional *vase* conformation: *δ* = 0.88 (*t*, 12H, ***J*** = 6.5 Hz, **CH_3_**CH_2_CH_2_), 1.23–1.32 (*m*, 32H, –**CH_2_**–), 2.16 (*bq*, 8H, CH**CH_2_**CH_2_), 4.04 (*s*, 24H, **CH_3_**OAr), 4.48 (*bt*, 4H, **CH**CH_2_CH_2_), 7.02 (*s*, 4H, Ar**H**_down_), 7.24 (*s*, 8H, **CH_3_**OAr**H_2_**) 7.48 (*s*, 4H, Ar**H**_up_). MALDI TOF–TOF: *m*/*z* = 1569 [*M*]^+^.

**Octa­hydroxy quinoxaline cavitand (6):** Cavitand **5** (0.03 mmol, 1 eq.) was dissolved in dry chloro­form (10 mL) and BBr_3_ (3.80 mmol, 120 eq.) was added dropwise under an Ar atmosphere. The mixture was stirred at 353 K for 24 h and H_2_O (30 mL) was added into a boiling solution. After cooling down to room temperature, chloro­form was removed and the yellow solid was sonicated with 1 *N* HCl, filtrated and dried under vacuum obtaining the final product **6** in qu­anti­tative yield.^1^H-NMR (300 MHz, DMSO-*d*_6_) – *vase* conformation: *δ* = 0.85 (*t*, 12H, ***J*** = 6.3 Hz, **CH_3_**CH_2_CH_2_), 1.12–1.37 (*m*, 32H, –**CH_2_**–), 2.37 (*bq*, 8H CH**CH_2_**CH_2_), 5.38 (*bt*, 4H, **CH**CH_2_CH_2_), 7.08 (*s*, 8H, **CH_3_**OAr**H_2_**), 7.69 (*s*, 4H, Ar**H**_down_), 7.84 (*s*, 4H, Ar**H**_up_), 9.94 (*s*, 8H, ArO**H**). MALDI TOF–TOF: *m*/*z* = 1457 [*M*]^+^.

**DeepQxCav (7):** Cavitand **6** (0.052 mmol, 1 eq.), ethyl­ene glycol di­tosyl­ate (0.52 mmol, 10 eq.), dry Cs_2_CO_3_ (0.63 mmol, 12 eq.) and dry DMF (5 mL) were added into an oven-dried microwave vessel under an Ar atmosphere and reacted under microwave irradiation at 393 K for 1.5 h. The reaction was quenched in water and extracted with DCM/H_2_O. The organic fractions were collected and dried over Na_2_SO_4_. After filtration the solvent was removed under reduced pressure and the crude was purified by flash chromatography. The final product **7** was obtained in 90% yield. **^1^H-NMR** (300 MHz, DMSO-*d*_6_) – *vase* conformation: *δ* = 0.86 (*s*, 12H, **CH_3_**CH_2_CH_2_), 1.20–1.48 (*m*, 32H, –**CH_2_**–), 2.40 (*bq*, 8H CH**CH_2_**CH_2_), 4.28–4.42 (*m*, 16H, ArO**CH_2_CH_2_**O), 5.48 (*t*, 4H, ***J*** = 7.6 Hz, **CH**CH_2_CH_2_), 7.20 (*s*, 8H, Ar**H_2_**), 7.74 (*s*, 4H, Ar**H**_down_), 7.89 (*s*, 4H, Ar**H**_up_). MALDI TOF–TOF: calculated for C_92_H_88_N_8_O_16_ [*M*]^+^ *m*/*z* = 1560.6318; found *m*/*z* = 1560.8065.

Prismatic, colourless single crystals of the title compound suitable for X-ray analysis were obtained by slow evaporation of a benzene solution.

## Refinement   

Crystal data, data collection and structure refinement details are summarized in Table 3 ▸.

The structure of the title compound was refined as a two-component twin with a BASF parameter of 0.572 (1). The last cycle of refinement was performed with a HKLF 5 dataset containing 12410 corrected reflections constructed from all observations involving domain 2.

A carbon atom (C23*B*) of one of the upper 1,4 dioxane rings was found to be disordered over two positions with occupancies of 0.547 (17) and 0.453 (17). The benzene guest was found disordered over two equally populated positions. For one of the two orientations (atoms C1*S*, C2*S*, C3*S* and their symmetry-generated analogues), the aromatic ring was modelled by fixing the bond distances to 1.380 (1) Å. The SIMU restraint (Sheldrick, 2015 ▸) was applied to atoms C4*S*–C7*S* of the second orientation.

The carbon-bound H atoms were placed in calculated positions and refined isotropically using a riding model with C—H ranging from 0.95 to 0.99 Å and *U*_iso_(H) set to 1.2–1.5*U*_eq_(C).

## Supplementary Material

Crystal structure: contains datablock(s) I. DOI: 10.1107/S2056989018017784/hb7791sup1.cif

Structure factors: contains datablock(s) I. DOI: 10.1107/S2056989018017784/hb7791Isup2.hkl

CCDC reference: 1885516

Additional supporting information:  crystallographic information; 3D view; checkCIF report

## Figures and Tables

**Figure 5:**
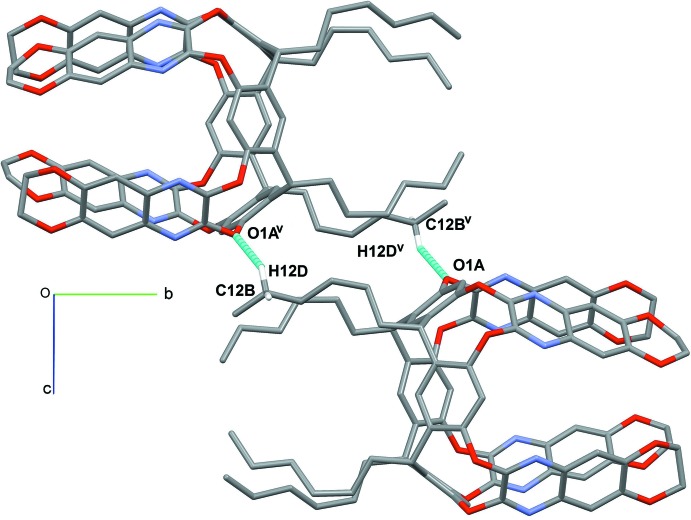
View of the relevant C—H⋯O hydrogen bonds (light-blue dotted lines) stabilizing the crystal structure of the title compound. Only the H atoms involved in the inter­actions are shown. The benzene guest has been omitted for clarity.

**Table 1:** Selected interatomic distances (Å)

C22*A*⋯C23*B*	4.053 (9)	C22*A*⋯C23*A* ^i^	8.181 (4)
C23*A*⋯C22*B* ^i^	3.757 (5)	C22*B*⋯C23*B* ^i^	4.664 (9)

Symmetry code: (i) 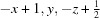.

**Table 2:** Hydrogen-bond geometry (Å, °) C*g*1 is the centroid of the ring C1*B*–C6*B*; C*g*2 is the centroid of the ring C14*A*–C17*A*/N1*A*/N2*A*.

*D*—H⋯*A*	*D*—H	H⋯*A*	*D*⋯*A*	*D*—H⋯*A*
C21*B*—H21*B*⋯O3*A* ^ii^	0.95	2.50	3.307 (3)	143
C18*A*—H18*A*⋯O3*A* ^ii^	0.95	2.40	3.302 (2)	158
C8*B* ^iii^—H8*B*2^iii^⋯C20*A*	0.99	2.71	3.693 (3)	170
C23*A*—H23*A*⋯C*g*1^iv^	0.99	2.88	3.530 (4)	124
C12*B* ^v^—H12*D* ^v^⋯O1*A*	0.99	2.71	3.414 (3)	128
C2*S*—H2*S*⋯C*g*2^i^	0.95	2.67	3.609 (3)	171

Symmetry codes: (i) 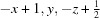; (ii) 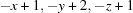; (iii) 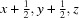; (iv) 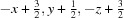; (v) 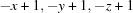.

**Table 3:** Experimental details

Crystal data	
Chemical formula	C_92_H_88_O_16_N_8_·C_6_H_6_
*M*_r_	1639.81
Crystal system, space group	Monoclinic, *C*2/*c*
Temperature (K)	190
*a*, *b*, *c* (Å)	19.173 (1), 20.756 (1), 21.771 (2)
β (°)	110.718 (2)
*V* (Å^3^)	8103.6 (9)
*Z*	4
Radiation type	Mo *K*α
μ (mm^−1^)	0.09
Crystal size (mm)	0.13 × 0.10 × 0.08

Data collection	
Diffractometer	Bruker APEXII CCD
Absorption correction	Multi-scan (*TWINABS*; Sheldrick, 2008 ▸)
*T*_min_, *T*_max_	0.671, 0.746
No. of measured, independent and observed [*I* > 2σ(*I*)] reflections	8326, 8326, 6387
*R*_int_	0.0
(sin θ/λ)_max_ (Å^−1^)	0.627

Refinement	
*R*[*F* ^2^ > 2σ(*F* ^2^)], *wR*(*F* ^2^), *S*	0.046, 0.119, 1.02
No. of reflections	8326
No. of parameters	573
No. of restraints	22
H-atom treatment	H-atom parameters constrained
Δρ_max_, Δρ_min_ (e Å^−3^)	0.35, −0.26

Computer programs: *APEX2* and *SAINT* (Bruker, 2008 ▸), *SIR97* (Altomare *et al.*, 1999 ▸), *SHELXL2014* (Sheldrick, 2015 ▸), *Mercury* (Macrae *et al.*, 2006 ▸), *WinGX* (Farrugia, 2012 ▸), *PARST* (Nardelli, 1995 ▸) and *publCIF* (Westrip, 2010 ▸).

## supplementary crystallographic information

### Crystal data

**Table:**

C_92_H_88_O_16_N_8_·C_6_H_6_	*F*(000) = 3464
*M**_r_* = 1639.81	*D*_x_ = 1.344 Mg m^−^^3^
Monoclinic, *C*2/*c*	Mo *K*α radiation, λ = 0.71073 Å
*a* = 19.173 (1) Å	Cell parameters from 1365 reflections
*b* = 20.756 (1) Å	θ = 1–26.5°
*c* = 21.771 (2) Å	µ = 0.09 mm^−^^1^
β = 110.718 (2)°	*T* = 190 K
*V* = 8103.6 (9) Å^3^	Prismatic, colourless
*Z* = 4	0.13 × 0.10 × 0.08 mm

### Data collection

**Table:**

Bruker APEXII CCD diffractometer	8326 independent reflections
Radiation source: fine-focus sealed tube	6387 reflections with *I* > 2σ(*I*)
Graphite monochromator	*R*_int_ = 0.0
ω scan	θ_max_ = 26.5°, θ_min_ = 1.5°
Absorption correction: multi-scan (*TWINABS*; Sheldrick, 2008)	*h* = −24→22
*T*_min_ = 0.671, *T*_max_ = 0.746	*k* = 0→25
8326 measured reflections	*l* = 0→27

### Refinement

**Table:**

Refinement on *F*^2^	Primary atom site location: structure-invariant direct methods
Least-squares matrix: full	Hydrogen site location: inferred from neighbouring sites
*R*[*F*^2^ > 2σ(*F*^2^)] = 0.046	H-atom parameters constrained
*wR*(*F*^2^) = 0.119	*w* = 1/[σ^2^(*F*_o_^2^) + (0.0582*P*)^2^ + 3.3175*P*] where *P* = (*F*_o_^2^ + 2*F*_c_^2^)/3
*S* = 1.02	(Δ/σ)_max_ = 0.001
8326 reflections	Δρ_max_ = 0.35 e Å^−^^3^
573 parameters	Δρ_min_ = −0.26 e Å^−^^3^
22 restraints	

### Special details

**Table:**

Geometry. All esds (except the esd in the dihedral angle between two l.s. planes) are estimated using the full covariance matrix. The cell esds are taken into account individually in the estimation of esds in distances, angles and torsion angles; correlations between esds in cell parameters are only used when they are defined by crystal symmetry. An approximate (isotropic) treatment of cell esds is used for estimating esds involving l.s. planes.
Refinement. Refined as a 2-component twin. 8600 Corrected reflections written to file twin4.hkl Reflections merged according to point-group 2/m Minimum and maximum apparent transmission: 0.671479 0.745373 Additional spherical absorption correction applied with mu*r = 0.2000 HKLF 5 dataset constructed from all observations involving domain 2 12410 Corrected reflections written to file twin5.hkl Reflections merged according to point-group 2/m Single reflections that also occur in composites omitted Minimum and maximum apparent transmission: 0.671267 0.745373 Additional spherical absorption correction applied with mu*r = 0.200

### Fractional atomic coordinates and isotropic or equivalent isotropic displacement parameters (Å^2^)

**Table:**

	*x*	*y*	*z*	*U*_iso_*/*U*_eq_	Occ. (<1)
C1S	0.4811 (3)	0.2252 (2)	0.2717 (2)	0.0445 (14)	0.5
H1S	0.4682	0.2647	0.2870	0.053*	0.5
C3S	0.4808 (3)	0.1094 (2)	0.2715 (2)	0.055 (2)	0.5
H3S	0.4673	0.0699	0.2863	0.066*	0.5
C2S	0.4622 (4)	0.16731 (19)	0.2928 (4)	0.070 (3)	0.5
H2S	0.4359	0.1673	0.3225	0.084*	0.5
C4S	0.5000	0.0994 (8)	0.2500	0.070 (2)	0.5
H4S	0.5000	0.0536	0.2500	0.083*	0.5
C7S	0.5000	0.2312 (6)	0.2500	0.070 (2)	0.5
H7S	0.5000	0.2769	0.2500	0.083*	0.5
C5S	0.4655 (7)	0.1331 (4)	0.2850 (6)	0.070 (2)	0.5
H5S	0.4407	0.1107	0.3093	0.083*	0.5
C6S	0.4664 (5)	0.1979 (4)	0.2852 (5)	0.070 (2)	0.5
H6S	0.4430	0.2207	0.3105	0.083*	0.5
N1A	0.59064 (9)	0.81836 (7)	0.54916 (8)	0.0263 (4)	
N2A	0.71761 (9)	0.81762 (8)	0.66567 (8)	0.0281 (4)	
O1A	0.59123 (7)	0.70686 (6)	0.55285 (6)	0.0256 (3)	
O2A	0.71490 (7)	0.70662 (6)	0.66212 (6)	0.0271 (3)	
O3A	0.57670 (9)	1.04593 (6)	0.56278 (7)	0.0364 (4)	
O4A	0.69554 (9)	1.04353 (7)	0.68594 (7)	0.0382 (4)	
C1A	0.47418 (11)	0.61563 (8)	0.62389 (8)	0.0213 (4)	
H1A	0.4779	0.5795	0.6517	0.026*	
C2A	0.53807 (10)	0.63498 (8)	0.61211 (9)	0.0216 (4)	
C3A	0.52994 (11)	0.68768 (9)	0.57033 (9)	0.0219 (4)	
C4A	0.46352 (11)	0.72085 (8)	0.54400 (9)	0.0217 (4)	
H4A	0.4598	0.7571	0.5164	0.026*	
C5A	0.40255 (10)	0.70036 (8)	0.55851 (9)	0.0214 (4)	
C6A	0.40516 (11)	0.64632 (8)	0.59717 (9)	0.0206 (4)	
C7A	0.61328 (10)	0.60203 (9)	0.64580 (9)	0.0222 (4)	
H7A	0.6444	0.6108	0.6183	0.027*	
C8A	0.60921 (11)	0.52876 (9)	0.65182 (9)	0.0259 (4)	
H8A1	0.6605	0.5116	0.6719	0.031*	
H8A2	0.5820	0.5186	0.6817	0.031*	
C9A	0.57066 (13)	0.49489 (10)	0.58614 (10)	0.0333 (5)	
H9A1	0.5875	0.5149	0.5525	0.040*	
H9A2	0.5162	0.5019	0.5725	0.040*	
C10A	0.58578 (13)	0.42232 (10)	0.58786 (10)	0.0352 (5)	
H10A	0.5548	0.4037	0.5450	0.042*	
H10B	0.6387	0.4157	0.5930	0.042*	
C11A	0.57026 (14)	0.38546 (10)	0.64198 (11)	0.0375 (5)	
H11A	0.5193	0.3960	0.6405	0.045*	
H11B	0.6058	0.3998	0.6851	0.045*	
C12A	0.57693 (14)	0.31275 (11)	0.63640 (12)	0.0432 (6)	
H12A	0.5380	0.2978	0.5953	0.052*	
H12B	0.6261	0.3026	0.6335	0.052*	
C13A	0.56887 (17)	0.27643 (12)	0.69372 (14)	0.0585 (7)	
H13A	0.5736	0.2301	0.6875	0.088*	
H13B	0.6079	0.2902	0.7345	0.088*	
H13C	0.5198	0.2854	0.6963	0.088*	
C14A	0.62121 (11)	0.76567 (9)	0.57833 (9)	0.0239 (4)	
C15A	0.68585 (11)	0.76556 (9)	0.63649 (9)	0.0251 (4)	
C16A	0.68381 (11)	0.87427 (9)	0.63890 (9)	0.0267 (4)	
C17A	0.62093 (11)	0.87498 (9)	0.57991 (9)	0.0261 (4)	
C18A	0.58733 (11)	0.93389 (9)	0.55450 (10)	0.0296 (5)	
H18A	0.5471	0.9351	0.5136	0.036*	
C19A	0.61237 (11)	0.98965 (10)	0.58853 (10)	0.0289 (4)	
C20A	0.67261 (11)	0.98849 (10)	0.64967 (10)	0.0288 (5)	
C21A	0.70941 (11)	0.93230 (10)	0.67279 (10)	0.0307 (5)	
H21A	0.7523	0.9323	0.7118	0.037*	
C22A	0.61161 (14)	1.10373 (10)	0.59622 (11)	0.0406 (6)	
H22A	0.5751	1.1395	0.5848	0.049*	
H22B	0.6536	1.1158	0.5821	0.049*	
C23A	0.63970 (14)	1.09286 (10)	0.66863 (12)	0.0407 (6)	
H23A	0.6611	1.1334	0.6916	0.049*	
H23B	0.5979	1.0799	0.6826	0.049*	
N1B	0.27919 (9)	0.84255 (8)	0.63477 (8)	0.0269 (4)	
N2B	0.36596 (9)	0.84062 (7)	0.55356 (8)	0.0268 (4)	
O1B	0.25211 (7)	0.73485 (6)	0.61013 (6)	0.0244 (3)	
O2B	0.33462 (7)	0.73349 (6)	0.53150 (6)	0.0238 (3)	
O3B	0.33383 (10)	1.06529 (7)	0.67816 (8)	0.0456 (4)	
O4B	0.42759 (10)	1.06135 (7)	0.60250 (9)	0.0456 (4)	
C1B	0.64056 (10)	0.61566 (9)	0.76929 (9)	0.0215 (4)	
H1B	0.6100	0.5791	0.7672	0.026*	
C2B	0.32793 (10)	0.64723 (8)	0.67067 (9)	0.0203 (4)	
C3B	0.28589 (10)	0.70178 (9)	0.66982 (9)	0.0217 (4)	
C4B	0.72827 (10)	0.72273 (9)	0.77536 (9)	0.0231 (4)	
H4B	0.7581	0.7597	0.7773	0.028*	
C5B	0.69772 (10)	0.68829 (9)	0.71774 (9)	0.0223 (4)	
C6B	0.65171 (10)	0.63515 (8)	0.71207 (9)	0.0209 (4)	
C7B	0.33514 (10)	0.62166 (8)	0.60727 (9)	0.0217 (4)	
H7B	0.2921	0.6402	0.5706	0.026*	
C8B	0.32661 (11)	0.54817 (8)	0.60047 (9)	0.0242 (4)	
H8B1	0.3699	0.5274	0.6340	0.029*	
H8B2	0.2812	0.5349	0.6088	0.029*	
C9B	0.32125 (13)	0.52490 (9)	0.53253 (10)	0.0305 (5)	
H9B1	0.3715	0.5264	0.5295	0.037*	
H9B2	0.2889	0.5549	0.4992	0.037*	
C10B	0.29031 (13)	0.45675 (9)	0.51620 (10)	0.0334 (5)	
H10C	0.2403	0.4554	0.5198	0.040*	
H10D	0.2839	0.4475	0.4699	0.040*	
C11B	0.33720 (13)	0.40362 (10)	0.55869 (11)	0.0360 (5)	
H11C	0.3424	0.4115	0.6050	0.043*	
H11D	0.3877	0.4048	0.5560	0.043*	
C12B	0.30369 (13)	0.33713 (9)	0.53828 (11)	0.0360 (5)	
H12C	0.2527	0.3364	0.5398	0.043*	
H12D	0.2995	0.3291	0.4923	0.043*	
C13B	0.34868 (16)	0.28340 (11)	0.58112 (14)	0.0519 (7)	
H13D	0.3242	0.2420	0.5655	0.078*	
H13E	0.3989	0.2830	0.5791	0.078*	
H13F	0.3520	0.2904	0.6266	0.078*	
C14B	0.28771 (10)	0.79144 (9)	0.60426 (9)	0.0232 (4)	
C15B	0.33131 (11)	0.79054 (9)	0.56320 (9)	0.0231 (4)	
C16B	0.35949 (11)	0.89601 (9)	0.58592 (9)	0.0268 (4)	
C17B	0.31538 (11)	0.89708 (9)	0.62631 (9)	0.0259 (4)	
C18B	0.30869 (13)	0.95474 (9)	0.65696 (10)	0.0325 (5)	
H18B	0.2790	0.9560	0.6839	0.039*	
C19B	0.34410 (12)	1.00920 (9)	0.64875 (10)	0.0305 (5)	
C20B	0.38977 (12)	1.00780 (9)	0.60987 (10)	0.0314 (5)	
C21B	0.39683 (13)	0.95212 (9)	0.57931 (11)	0.0336 (5)	
H21B	0.4274	0.9514	0.5532	0.040*	
C22B	0.3660 (2)	1.11976 (12)	0.66256 (19)	0.0798 (11)	0.547 (17)
H22C	0.3267	1.1423	0.6267	0.096*	0.547 (17)
H22D	0.3803	1.1486	0.7013	0.096*	0.547 (17)
C23B	0.4260 (5)	1.1140 (3)	0.6441 (6)	0.048 (2)	0.547 (17)
H23C	0.4708	1.1108	0.6845	0.058*	0.547 (17)
H23D	0.4305	1.1543	0.6215	0.058*	0.547 (17)
C22C	0.3660 (2)	1.11976 (12)	0.66256 (19)	0.0798 (11)	0.453 (17)
H22E	0.4070	1.1330	0.7030	0.096*	0.453 (17)
H22F	0.3280	1.1544	0.6525	0.096*	0.453 (17)
C23C	0.3952 (7)	1.1197 (3)	0.6112 (6)	0.040 (2)	0.453 (17)
H23E	0.3545	1.1306	0.5696	0.048*	0.453 (17)
H23F	0.4333	1.1541	0.6200	0.048*	0.453 (17)

### Atomic displacement parameters (Å^2^)

**Table:**

	*U*^11^	*U*^22^	*U*^33^	*U*^12^	*U*^13^	*U*^23^
C1S	0.059 (4)	0.039 (3)	0.060 (4)	−0.004 (3)	0.052 (3)	−0.005 (3)
C3S	0.046 (4)	0.037 (4)	0.062 (5)	−0.005 (3)	−0.005 (3)	0.022 (4)
C2S	0.109 (7)	0.050 (5)	0.085 (5)	−0.007 (5)	0.078 (5)	0.015 (4)
C4S	0.066 (4)	0.074 (4)	0.078 (4)	0.000	0.036 (3)	0.000
C7S	0.066 (4)	0.074 (4)	0.078 (4)	0.000	0.036 (3)	0.000
C5S	0.066 (4)	0.074 (4)	0.078 (4)	0.000	0.036 (3)	0.000
C6S	0.066 (4)	0.074 (4)	0.078 (4)	0.000	0.036 (3)	0.000
N1A	0.0287 (9)	0.0273 (9)	0.0243 (8)	−0.0025 (7)	0.0112 (7)	0.0023 (7)
N2A	0.0268 (9)	0.0328 (10)	0.0262 (9)	−0.0035 (7)	0.0113 (7)	0.0045 (7)
O1A	0.0303 (8)	0.0260 (7)	0.0256 (7)	−0.0028 (6)	0.0161 (6)	−0.0011 (6)
O2A	0.0296 (8)	0.0298 (7)	0.0266 (7)	0.0039 (6)	0.0156 (6)	0.0052 (6)
O3A	0.0394 (9)	0.0261 (8)	0.0401 (9)	−0.0004 (6)	0.0095 (7)	0.0005 (6)
O4A	0.0395 (9)	0.0321 (8)	0.0395 (9)	−0.0087 (7)	0.0099 (7)	−0.0075 (7)
C1A	0.0284 (10)	0.0180 (9)	0.0177 (9)	0.0004 (8)	0.0083 (8)	−0.0007 (7)
C2A	0.0268 (10)	0.0189 (9)	0.0189 (9)	0.0003 (8)	0.0078 (8)	−0.0033 (7)
C3A	0.0254 (10)	0.0223 (9)	0.0193 (9)	−0.0027 (8)	0.0096 (8)	−0.0042 (7)
C4A	0.0305 (11)	0.0180 (9)	0.0167 (9)	−0.0001 (8)	0.0084 (8)	0.0013 (7)
C5A	0.0234 (10)	0.0201 (9)	0.0189 (9)	0.0010 (8)	0.0051 (8)	−0.0029 (7)
C6A	0.0248 (10)	0.0184 (9)	0.0185 (9)	−0.0010 (8)	0.0077 (8)	−0.0042 (7)
C7A	0.0254 (10)	0.0232 (9)	0.0203 (9)	0.0035 (8)	0.0108 (8)	−0.0012 (7)
C8A	0.0306 (11)	0.0236 (10)	0.0232 (10)	0.0050 (8)	0.0093 (8)	−0.0013 (8)
C9A	0.0461 (13)	0.0283 (11)	0.0260 (10)	0.0032 (10)	0.0134 (9)	−0.0042 (9)
C10A	0.0439 (13)	0.0301 (11)	0.0316 (11)	0.0020 (10)	0.0135 (10)	−0.0079 (9)
C11A	0.0441 (14)	0.0325 (12)	0.0363 (12)	0.0013 (10)	0.0146 (10)	−0.0039 (10)
C12A	0.0467 (15)	0.0354 (13)	0.0493 (14)	0.0012 (11)	0.0193 (12)	0.0000 (11)
C13A	0.071 (2)	0.0461 (15)	0.0641 (18)	0.0039 (14)	0.0307 (15)	0.0120 (13)
C14A	0.0248 (10)	0.0267 (10)	0.0242 (10)	−0.0030 (8)	0.0136 (8)	0.0007 (8)
C15A	0.0258 (11)	0.0306 (11)	0.0237 (10)	−0.0004 (9)	0.0146 (8)	0.0035 (8)
C16A	0.0248 (11)	0.0306 (11)	0.0264 (10)	−0.0040 (8)	0.0111 (8)	0.0029 (8)
C17A	0.0269 (11)	0.0288 (10)	0.0250 (10)	−0.0041 (8)	0.0120 (8)	0.0028 (8)
C18A	0.0294 (11)	0.0308 (11)	0.0261 (10)	−0.0028 (9)	0.0066 (9)	0.0028 (9)
C19A	0.0288 (11)	0.0274 (11)	0.0332 (11)	−0.0013 (9)	0.0141 (9)	0.0039 (9)
C20A	0.0279 (11)	0.0320 (11)	0.0283 (10)	−0.0098 (9)	0.0122 (9)	−0.0026 (9)
C21A	0.0258 (11)	0.0361 (12)	0.0280 (10)	−0.0090 (9)	0.0067 (8)	0.0033 (9)
C22A	0.0460 (14)	0.0280 (12)	0.0467 (14)	−0.0066 (10)	0.0152 (11)	−0.0023 (10)
C23A	0.0468 (14)	0.0305 (12)	0.0469 (14)	−0.0055 (10)	0.0193 (12)	−0.0068 (10)
N1B	0.0298 (9)	0.0257 (9)	0.0266 (9)	0.0036 (7)	0.0116 (7)	0.0022 (7)
N2B	0.0333 (10)	0.0227 (8)	0.0276 (9)	0.0037 (7)	0.0146 (8)	0.0016 (7)
O1B	0.0257 (7)	0.0234 (7)	0.0218 (7)	0.0003 (6)	0.0055 (5)	0.0030 (5)
O2B	0.0258 (7)	0.0218 (7)	0.0213 (6)	0.0045 (6)	0.0054 (5)	0.0000 (5)
O3B	0.0694 (12)	0.0234 (8)	0.0560 (10)	−0.0021 (8)	0.0370 (9)	−0.0082 (7)
O4B	0.0635 (12)	0.0229 (8)	0.0643 (11)	−0.0067 (7)	0.0399 (9)	−0.0047 (7)
C1B	0.0216 (10)	0.0189 (9)	0.0245 (9)	0.0012 (7)	0.0087 (8)	0.0009 (7)
C2B	0.0200 (10)	0.0192 (9)	0.0234 (9)	−0.0052 (8)	0.0099 (8)	−0.0014 (8)
C3B	0.0193 (10)	0.0217 (9)	0.0221 (9)	−0.0030 (8)	0.0050 (7)	0.0010 (8)
C4B	0.0200 (10)	0.0218 (9)	0.0271 (10)	0.0007 (8)	0.0080 (8)	0.0021 (8)
C5B	0.0218 (10)	0.0252 (10)	0.0220 (9)	0.0064 (8)	0.0104 (8)	0.0054 (8)
C6B	0.0197 (10)	0.0213 (9)	0.0209 (9)	0.0054 (7)	0.0063 (7)	0.0007 (7)
C7B	0.0232 (10)	0.0205 (9)	0.0204 (9)	−0.0003 (8)	0.0066 (8)	0.0005 (7)
C8B	0.0286 (11)	0.0209 (9)	0.0238 (10)	−0.0042 (8)	0.0104 (8)	−0.0015 (8)
C9B	0.0413 (13)	0.0249 (10)	0.0245 (10)	−0.0014 (9)	0.0105 (9)	−0.0040 (8)
C10B	0.0369 (13)	0.0298 (11)	0.0306 (11)	−0.0025 (9)	0.0082 (9)	−0.0078 (9)
C11B	0.0395 (13)	0.0283 (11)	0.0374 (12)	−0.0021 (10)	0.0101 (10)	−0.0040 (9)
C12B	0.0415 (14)	0.0275 (11)	0.0438 (13)	−0.0033 (9)	0.0209 (11)	−0.0044 (9)
C13B	0.0600 (18)	0.0340 (13)	0.0639 (17)	0.0004 (12)	0.0245 (14)	0.0016 (12)
C14B	0.0208 (10)	0.0237 (10)	0.0227 (9)	0.0047 (8)	0.0049 (8)	0.0031 (8)
C15B	0.0254 (11)	0.0201 (9)	0.0199 (9)	0.0046 (8)	0.0033 (8)	0.0009 (7)
C16B	0.0322 (11)	0.0230 (10)	0.0263 (10)	0.0061 (8)	0.0118 (9)	0.0027 (8)
C17B	0.0279 (11)	0.0235 (10)	0.0259 (10)	0.0043 (8)	0.0091 (8)	0.0030 (8)
C18B	0.0413 (13)	0.0290 (11)	0.0331 (11)	0.0050 (9)	0.0203 (10)	0.0009 (9)
C19B	0.0403 (13)	0.0226 (10)	0.0293 (10)	0.0056 (9)	0.0131 (9)	−0.0009 (8)
C20B	0.0358 (12)	0.0242 (10)	0.0346 (11)	−0.0004 (9)	0.0131 (9)	0.0034 (9)
C21B	0.0400 (13)	0.0286 (11)	0.0406 (12)	0.0014 (9)	0.0247 (11)	0.0019 (9)
C22B	0.137 (3)	0.0263 (14)	0.119 (3)	−0.0099 (17)	0.098 (3)	−0.0105 (16)
C23B	0.055 (4)	0.027 (3)	0.066 (6)	−0.008 (3)	0.024 (4)	−0.013 (3)
C22C	0.137 (3)	0.0263 (14)	0.119 (3)	−0.0099 (17)	0.098 (3)	−0.0105 (16)
C23C	0.055 (5)	0.018 (3)	0.046 (5)	−0.002 (3)	0.016 (4)	0.000 (3)

### Geometric parameters (Å, º)

**Table:**

C1S—C1S^i^	1.3798 (10)	C22A—C23A	1.492 (3)
C1S—C2S	1.3803 (10)	C22A—H22A	0.9900
C1S—H1S	0.9500	C22A—H22B	0.9900
C3S—C3S^i^	1.3800 (10)	C23A—H23A	0.9900
C3S—C2S	1.3804 (10)	C23A—H23B	0.9900
C3S—H3S	0.9500	N1B—C14B	1.292 (2)
C2S—H2S	0.9500	N1B—C17B	1.374 (3)
C4S—C5S^i^	1.365 (13)	N2B—C15B	1.290 (2)
C4S—C5S	1.365 (13)	N2B—C16B	1.377 (2)
C4S—H4S	0.9500	O1B—C14B	1.387 (2)
C7S—C6S	1.351 (10)	O1B—C3B	1.408 (2)
C7S—C6S^i^	1.351 (10)	O2B—C15B	1.383 (2)
C7S—H7S	0.9500	O3B—C19B	1.376 (2)
C5S—C6S	1.345 (11)	O3B—C22C	1.386 (3)
C5S—H5S	0.9500	O3B—C22B	1.386 (3)
C6S—H6S	0.9500	O4B—C20B	1.367 (2)
N1A—C14A	1.296 (2)	O4B—C23C	1.404 (7)
N1A—C17A	1.375 (2)	O4B—C23B	1.427 (6)
N2A—C15A	1.292 (2)	C1B—C2B^ii^	1.394 (3)
N2A—C16A	1.369 (2)	C1B—C6B	1.396 (3)
O1A—C14A	1.379 (2)	C1B—H1B	0.9500
O1A—C3A	1.414 (2)	C2B—C3B	1.386 (3)
O2A—C15A	1.378 (2)	C2B—C1B^ii^	1.394 (3)
O2A—C5B	1.415 (2)	C2B—C7B	1.530 (2)
O3A—C19A	1.369 (2)	C3B—C4B^ii^	1.385 (3)
O3A—C22A	1.439 (2)	C4B—C5B	1.381 (3)
O4A—C20A	1.370 (2)	C4B—C3B^ii^	1.384 (3)
O4A—C23A	1.432 (3)	C4B—H4B	0.9500
C1A—C2A	1.396 (3)	C5B—C6B	1.390 (3)
C1A—C6A	1.397 (3)	C7B—C8B	1.536 (2)
C1A—H1A	0.9500	C7B—H7B	1.0000
C2A—C3A	1.396 (2)	C8B—C9B	1.524 (3)
C2A—C7A	1.529 (3)	C8B—H8B1	0.9900
C3A—C4A	1.381 (3)	C8B—H8B2	0.9900
C4A—C5A	1.382 (3)	C9B—C10B	1.526 (3)
C4A—H4A	0.9500	C9B—H9B1	0.9900
C5A—C6A	1.393 (3)	C9B—H9B2	0.9900
C5A—O2B	1.405 (2)	C10B—C11B	1.514 (3)
C6A—C7B	1.524 (3)	C10B—H10C	0.9900
C7A—C8A	1.531 (2)	C10B—H10D	0.9900
C7A—C6B	1.531 (2)	C11B—C12B	1.521 (3)
C7A—H7A	1.0000	C11B—H11C	0.9900
C8A—C9A	1.529 (3)	C11B—H11D	0.9900
C8A—H8A1	0.9900	C12B—C13B	1.513 (3)
C8A—H8A2	0.9900	C12B—H12C	0.9900
C9A—C10A	1.532 (3)	C12B—H12D	0.9900
C9A—H9A1	0.9900	C13B—H13D	0.9800
C9A—H9A2	0.9900	C13B—H13E	0.9800
C10A—C11A	1.520 (3)	C13B—H13F	0.9800
C10A—H10A	0.9900	C14B—C15B	1.424 (3)
C10A—H10B	0.9900	C16B—C21B	1.401 (3)
C11A—C12A	1.523 (3)	C16B—C17B	1.419 (3)
C11A—H11A	0.9900	C17B—C18B	1.398 (3)
C11A—H11B	0.9900	C18B—C19B	1.362 (3)
C12A—C13A	1.512 (3)	C18B—H18B	0.9500
C12A—H12A	0.9900	C19B—C20B	1.417 (3)
C12A—H12B	0.9900	C20B—C21B	1.364 (3)
C13A—H13A	0.9800	C21B—H21B	0.9500
C13A—H13B	0.9800	C22B—C23B	1.351 (7)
C13A—H13C	0.9800	C22B—H22C	0.9900
C14A—C15A	1.424 (3)	C22B—H22D	0.9900
C16A—C21A	1.407 (3)	C23B—H23C	0.9900
C16A—C17A	1.417 (3)	C23B—H23D	0.9900
C17A—C18A	1.401 (3)	C22C—C23C	1.418 (8)
C18A—C19A	1.366 (3)	C22C—H22E	0.9900
C18A—H18A	0.9500	C22C—H22F	0.9900
C19A—C20A	1.421 (3)	C23C—H23E	0.9900
C20A—C21A	1.364 (3)	C23C—H23F	0.9900
C21A—H21A	0.9500		
			
C22*A*···C23*B*	4.053 (9)	C22*A*···C23*A*^i^	8.181 (4)
C23*A*···C22*B*^i^	3.757 (5)	C22*B*···C23*B*^i^	4.664 (9)
			
C1S^i^—C1S—C2S	119.4 (2)	O4A—C23A—H23B	109.6
C1S^i^—C1S—H1S	120.3	C22A—C23A—H23B	109.6
C2S—C1S—H1S	120.3	H23A—C23A—H23B	108.2
C3S^i^—C3S—C2S	119.4 (2)	C14B—N1B—C17B	116.48 (17)
C3S^i^—C3S—H3S	120.3	C15B—N2B—C16B	116.41 (17)
C2S—C3S—H3S	120.3	C14B—O1B—C3B	114.65 (14)
C1S—C2S—C3S	121.1 (4)	C15B—O2B—C5A	114.19 (14)
C1S—C2S—H2S	119.4	C19B—O3B—C22C	115.27 (19)
C3S—C2S—H2S	119.4	C19B—O3B—C22B	115.27 (19)
C5S^i^—C4S—C5S	118.2 (16)	C20B—O4B—C23C	114.1 (4)
C5S^i^—C4S—H4S	120.9	C20B—O4B—C23B	114.6 (3)
C5S—C4S—H4S	120.9	C2B^ii^—C1B—C6B	123.22 (17)
C6S—C7S—C6S^i^	118.6 (13)	C2B^ii^—C1B—H1B	118.4
C6S—C7S—H7S	120.7	C6B—C1B—H1B	118.4
C6S^i^—C7S—H7S	120.7	C3B—C2B—C1B^ii^	116.97 (16)
C6S—C5S—C4S	120.5 (11)	C3B—C2B—C7B	120.59 (16)
C6S—C5S—H5S	119.8	C1B^ii^—C2B—C7B	122.36 (16)
C4S—C5S—H5S	119.8	C4B^ii^—C3B—C2B	122.38 (17)
C5S—C6S—C7S	121.1 (10)	C4B^ii^—C3B—O1B	118.42 (16)
C5S—C6S—H6S	119.4	C2B—C3B—O1B	119.06 (16)
C7S—C6S—H6S	119.4	C5B—C4B—C3B^ii^	118.09 (17)
C14A—N1A—C17A	116.28 (17)	C5B—C4B—H4B	121.0
C15A—N2A—C16A	116.06 (17)	C3B^ii^—C4B—H4B	121.0
C14A—O1A—C3A	114.12 (14)	C4B—C5B—C6B	122.90 (17)
C15A—O2A—C5B	113.73 (14)	C4B—C5B—O2A	119.06 (17)
C19A—O3A—C22A	115.36 (17)	C6B—C5B—O2A	118.03 (16)
C20A—O4A—C23A	112.77 (16)	C5B—C6B—C1B	116.30 (17)
C2A—C1A—C6A	123.77 (17)	C5B—C6B—C7A	121.24 (16)
C2A—C1A—H1A	118.1	C1B—C6B—C7A	122.41 (17)
C6A—C1A—H1A	118.1	C6A—C7B—C2B	112.28 (14)
C3A—C2A—C1A	116.25 (17)	C6A—C7B—C8B	112.88 (15)
C3A—C2A—C7A	122.16 (17)	C2B—C7B—C8B	113.02 (15)
C1A—C2A—C7A	121.54 (16)	C6A—C7B—H7B	106.0
C4A—C3A—C2A	122.34 (18)	C2B—C7B—H7B	106.0
C4A—C3A—O1A	118.68 (16)	C8B—C7B—H7B	106.0
C2A—C3A—O1A	118.95 (17)	C9B—C8B—C7B	112.12 (15)
C3A—C4A—C5A	118.82 (17)	C9B—C8B—H8B1	109.2
C3A—C4A—H4A	120.6	C7B—C8B—H8B1	109.2
C5A—C4A—H4A	120.6	C9B—C8B—H8B2	109.2
C4A—C5A—C6A	122.26 (17)	C7B—C8B—H8B2	109.2
C4A—C5A—O2B	119.19 (16)	H8B1—C8B—H8B2	107.9
C6A—C5A—O2B	118.50 (16)	C8B—C9B—C10B	114.01 (17)
C5A—C6A—C1A	116.44 (17)	C8B—C9B—H9B1	108.7
C5A—C6A—C7B	120.83 (17)	C10B—C9B—H9B1	108.7
C1A—C6A—C7B	122.70 (16)	C8B—C9B—H9B2	108.7
C2A—C7A—C8A	114.68 (16)	C10B—C9B—H9B2	108.7
C2A—C7A—C6B	108.00 (14)	H9B1—C9B—H9B2	107.6
C8A—C7A—C6B	112.80 (15)	C11B—C10B—C9B	115.78 (18)
C2A—C7A—H7A	107.0	C11B—C10B—H10C	108.3
C8A—C7A—H7A	107.0	C9B—C10B—H10C	108.3
C6B—C7A—H7A	107.0	C11B—C10B—H10D	108.3
C9A—C8A—C7A	113.53 (16)	C9B—C10B—H10D	108.3
C9A—C8A—H8A1	108.9	H10C—C10B—H10D	107.4
C7A—C8A—H8A1	108.9	C10B—C11B—C12B	112.61 (18)
C9A—C8A—H8A2	108.9	C10B—C11B—H11C	109.1
C7A—C8A—H8A2	108.9	C12B—C11B—H11C	109.1
H8A1—C8A—H8A2	107.7	C10B—C11B—H11D	109.1
C8A—C9A—C10A	113.86 (17)	C12B—C11B—H11D	109.1
C8A—C9A—H9A1	108.8	H11C—C11B—H11D	107.8
C10A—C9A—H9A1	108.8	C13B—C12B—C11B	113.51 (19)
C8A—C9A—H9A2	108.8	C13B—C12B—H12C	108.9
C10A—C9A—H9A2	108.8	C11B—C12B—H12C	108.9
H9A1—C9A—H9A2	107.7	C13B—C12B—H12D	108.9
C11A—C10A—C9A	115.26 (17)	C11B—C12B—H12D	108.9
C11A—C10A—H10A	108.5	H12C—C12B—H12D	107.7
C9A—C10A—H10A	108.5	C12B—C13B—H13D	109.5
C11A—C10A—H10B	108.5	C12B—C13B—H13E	109.5
C9A—C10A—H10B	108.5	H13D—C13B—H13E	109.5
H10A—C10A—H10B	107.5	C12B—C13B—H13F	109.5
C10A—C11A—C12A	113.08 (18)	H13D—C13B—H13F	109.5
C10A—C11A—H11A	109.0	H13E—C13B—H13F	109.5
C12A—C11A—H11A	109.0	N1B—C14B—O1B	119.48 (17)
C10A—C11A—H11B	109.0	N1B—C14B—C15B	122.85 (18)
C12A—C11A—H11B	109.0	O1B—C14B—C15B	117.66 (16)
H11A—C11A—H11B	107.8	N2B—C15B—O2B	119.43 (17)
C13A—C12A—C11A	113.1 (2)	N2B—C15B—C14B	122.83 (17)
C13A—C12A—H12A	109.0	O2B—C15B—C14B	117.72 (16)
C11A—C12A—H12A	109.0	N2B—C16B—C21B	119.98 (18)
C13A—C12A—H12B	109.0	N2B—C16B—C17B	120.76 (18)
C11A—C12A—H12B	109.0	C21B—C16B—C17B	119.26 (18)
H12A—C12A—H12B	107.8	N1B—C17B—C18B	120.36 (18)
C12A—C13A—H13A	109.5	N1B—C17B—C16B	120.66 (17)
C12A—C13A—H13B	109.5	C18B—C17B—C16B	118.98 (18)
H13A—C13A—H13B	109.5	C19B—C18B—C17B	120.89 (19)
C12A—C13A—H13C	109.5	C19B—C18B—H18B	119.6
H13A—C13A—H13C	109.5	C17B—C18B—H18B	119.6
H13B—C13A—H13C	109.5	C18B—C19B—O3B	118.75 (19)
N1A—C14A—O1A	119.86 (17)	C18B—C19B—C20B	120.21 (18)
N1A—C14A—C15A	122.50 (18)	O3B—C19B—C20B	121.04 (18)
O1A—C14A—C15A	117.63 (17)	C21B—C20B—O4B	118.90 (19)
N2A—C15A—O2A	119.40 (17)	C21B—C20B—C19B	119.84 (19)
N2A—C15A—C14A	123.12 (18)	O4B—C20B—C19B	121.25 (18)
O2A—C15A—C14A	117.48 (18)	C20B—C21B—C16B	120.8 (2)
N2A—C16A—C21A	119.15 (18)	C20B—C21B—H21B	119.6
N2A—C16A—C17A	121.17 (18)	C16B—C21B—H21B	119.6
C21A—C16A—C17A	119.57 (19)	C23B—C22B—O3B	120.0 (4)
N1A—C17A—C18A	119.83 (17)	C23B—C22B—H22C	107.3
N1A—C17A—C16A	120.64 (18)	O3B—C22B—H22C	107.3
C18A—C17A—C16A	119.46 (18)	C23B—C22B—H22D	107.3
C19A—C18A—C17A	120.04 (19)	O3B—C22B—H22D	107.3
C19A—C18A—H18A	120.0	H22C—C22B—H22D	106.9
C17A—C18A—H18A	120.0	C22B—C23B—O4B	118.0 (5)
C18A—C19A—O3A	118.15 (18)	C22B—C23B—H23C	107.8
C18A—C19A—C20A	120.45 (19)	O4B—C23B—H23C	107.8
O3A—C19A—C20A	121.38 (18)	C22B—C23B—H23D	107.8
C21A—C20A—O4A	118.81 (18)	O4B—C23B—H23D	107.8
C21A—C20A—C19A	120.21 (18)	H23C—C23B—H23D	107.2
O4A—C20A—C19A	120.97 (19)	O3B—C22C—C23C	121.8 (4)
C20A—C21A—C16A	119.97 (19)	O3B—C22C—H22E	106.9
C20A—C21A—H21A	120.0	C23C—C22C—H22E	106.9
C16A—C21A—H21A	120.0	O3B—C22C—H22F	106.9
O3A—C22A—C23A	109.75 (18)	C23C—C22C—H22F	106.9
O3A—C22A—H22A	109.7	H22E—C22C—H22F	106.7
C23A—C22A—H22A	109.7	O4B—C23C—C22C	115.0 (5)
O3A—C22A—H22B	109.7	O4B—C23C—H23E	108.5
C23A—C22A—H22B	109.7	C22C—C23C—H23E	108.5
H22A—C22A—H22B	108.2	O4B—C23C—H23F	108.5
O4A—C23A—C22A	110.06 (19)	C22C—C23C—H23F	108.5
O4A—C23A—H23A	109.6	H23E—C23C—H23F	107.5
C22A—C23A—H23A	109.6		
			
C1S^i^—C1S—C2S—C3S	0.4 (11)	C7B—C2B—C3B—O1B	−2.4 (3)
C3S^i^—C3S—C2S—C1S	0.2 (11)	C14B—O1B—C3B—C4B^ii^	80.5 (2)
C5S^i^—C4S—C5S—C6S	−0.6 (8)	C14B—O1B—C3B—C2B	−103.66 (19)
C4S—C5S—C6S—C7S	1.3 (16)	C3B^ii^—C4B—C5B—C6B	2.2 (3)
C6S^i^—C7S—C6S—C5S	−0.6 (8)	C3B^ii^—C4B—C5B—O2A	−176.44 (16)
C6A—C1A—C2A—C3A	−0.8 (3)	C15A—O2A—C5B—C4B	−67.5 (2)
C6A—C1A—C2A—C7A	176.41 (16)	C15A—O2A—C5B—C6B	113.82 (18)
C1A—C2A—C3A—C4A	2.6 (3)	C4B—C5B—C6B—C1B	−3.3 (3)
C7A—C2A—C3A—C4A	−174.63 (16)	O2A—C5B—C6B—C1B	175.37 (15)
C1A—C2A—C3A—O1A	−175.70 (15)	C4B—C5B—C6B—C7A	174.37 (17)
C7A—C2A—C3A—O1A	7.1 (2)	O2A—C5B—C6B—C7A	−7.0 (2)
C14A—O1A—C3A—C4A	69.6 (2)	C2B^ii^—C1B—C6B—C5B	0.9 (3)
C14A—O1A—C3A—C2A	−112.00 (18)	C2B^ii^—C1B—C6B—C7A	−176.66 (16)
C2A—C3A—C4A—C5A	−1.3 (3)	C2A—C7A—C6B—C5B	−89.2 (2)
O1A—C3A—C4A—C5A	177.00 (15)	C8A—C7A—C6B—C5B	143.00 (17)
C3A—C4A—C5A—C6A	−1.9 (3)	C2A—C7A—C6B—C1B	88.3 (2)
C3A—C4A—C5A—O2B	−179.48 (15)	C8A—C7A—C6B—C1B	−39.5 (2)
C4A—C5A—C6A—C1A	3.5 (3)	C5A—C6A—C7B—C2B	−95.5 (2)
O2B—C5A—C6A—C1A	−178.92 (15)	C1A—C6A—C7B—C2B	86.5 (2)
C4A—C5A—C6A—C7B	−174.61 (16)	C5A—C6A—C7B—C8B	135.34 (17)
O2B—C5A—C6A—C7B	3.0 (2)	C1A—C6A—C7B—C8B	−42.7 (2)
C2A—C1A—C6A—C5A	−2.1 (3)	C3B—C2B—C7B—C6A	94.9 (2)
C2A—C1A—C6A—C7B	175.98 (16)	C1B^ii^—C2B—C7B—C6A	−88.4 (2)
C3A—C2A—C7A—C8A	−143.06 (17)	C3B—C2B—C7B—C8B	−135.96 (18)
C1A—C2A—C7A—C8A	39.9 (2)	C1B^ii^—C2B—C7B—C8B	40.7 (2)
C3A—C2A—C7A—C6B	90.2 (2)	C6A—C7B—C8B—C9B	−60.6 (2)
C1A—C2A—C7A—C6B	−86.8 (2)	C2B—C7B—C8B—C9B	170.66 (16)
C2A—C7A—C8A—C9A	55.5 (2)	C7B—C8B—C9B—C10B	−162.95 (17)
C6B—C7A—C8A—C9A	179.72 (16)	C8B—C9B—C10B—C11B	−64.0 (3)
C7A—C8A—C9A—C10A	163.16 (18)	C9B—C10B—C11B—C12B	−178.55 (18)
C8A—C9A—C10A—C11A	52.5 (3)	C10B—C11B—C12B—C13B	−178.6 (2)
C9A—C10A—C11A—C12A	172.89 (19)	C17B—N1B—C14B—O1B	−179.37 (16)
C10A—C11A—C12A—C13A	174.4 (2)	C17B—N1B—C14B—C15B	−0.7 (3)
C17A—N1A—C14A—O1A	175.95 (16)	C3B—O1B—C14B—N1B	−76.9 (2)
C17A—N1A—C14A—C15A	−4.3 (3)	C3B—O1B—C14B—C15B	104.30 (19)
C3A—O1A—C14A—N1A	−82.1 (2)	C16B—N2B—C15B—O2B	179.13 (16)
C3A—O1A—C14A—C15A	98.2 (2)	C16B—N2B—C15B—C14B	0.7 (3)
C16A—N2A—C15A—O2A	−177.38 (16)	C5A—O2B—C15B—N2B	75.9 (2)
C16A—N2A—C15A—C14A	2.5 (3)	C5A—O2B—C15B—C14B	−105.54 (18)
C5B—O2A—C15A—N2A	76.9 (2)	N1B—C14B—C15B—N2B	0.4 (3)
C5B—O2A—C15A—C14A	−103.00 (19)	O1B—C14B—C15B—N2B	179.08 (16)
N1A—C14A—C15A—N2A	2.1 (3)	N1B—C14B—C15B—O2B	−178.14 (16)
O1A—C14A—C15A—N2A	−178.18 (16)	O1B—C14B—C15B—O2B	0.6 (2)
N1A—C14A—C15A—O2A	−178.04 (16)	C15B—N2B—C16B—C21B	178.51 (19)
O1A—C14A—C15A—O2A	1.7 (3)	C15B—N2B—C16B—C17B	−1.3 (3)
C15A—N2A—C16A—C21A	171.71 (18)	C14B—N1B—C17B—C18B	179.37 (18)
C15A—N2A—C16A—C17A	−4.4 (3)	C14B—N1B—C17B—C16B	0.0 (3)
C14A—N1A—C17A—C18A	−174.73 (18)	N2B—C16B—C17B—N1B	1.0 (3)
C14A—N1A—C17A—C16A	2.2 (3)	C21B—C16B—C17B—N1B	−178.80 (18)
N2A—C16A—C17A—N1A	2.2 (3)	N2B—C16B—C17B—C18B	−178.35 (18)
C21A—C16A—C17A—N1A	−173.92 (17)	C21B—C16B—C17B—C18B	1.8 (3)
N2A—C16A—C17A—C18A	179.21 (18)	N1B—C17B—C18B—C19B	−179.76 (19)
C21A—C16A—C17A—C18A	3.1 (3)	C16B—C17B—C18B—C19B	−0.4 (3)
N1A—C17A—C18A—C19A	173.10 (18)	C17B—C18B—C19B—O3B	177.87 (19)
C16A—C17A—C18A—C19A	−3.9 (3)	C17B—C18B—C19B—C20B	−1.3 (3)
C17A—C18A—C19A—O3A	−178.31 (18)	C22C—O3B—C19B—C18B	−174.2 (3)
C17A—C18A—C19A—C20A	0.2 (3)	C22B—O3B—C19B—C18B	−174.2 (3)
C22A—O3A—C19A—C18A	−172.14 (19)	C22C—O3B—C19B—C20B	5.0 (3)
C22A—O3A—C19A—C20A	9.4 (3)	C22B—O3B—C19B—C20B	5.0 (3)
C23A—O4A—C20A—C21A	−160.78 (19)	C23C—O4B—C20B—C21B	155.0 (6)
C23A—O4A—C20A—C19A	20.2 (3)	C23B—O4B—C20B—C21B	−170.7 (6)
C18A—C19A—C20A—C21A	4.6 (3)	C23C—O4B—C20B—C19B	−25.4 (6)
O3A—C19A—C20A—C21A	−176.99 (18)	C23B—O4B—C20B—C19B	8.9 (6)
C18A—C19A—C20A—O4A	−176.38 (18)	C18B—C19B—C20B—C21B	1.6 (3)
O3A—C19A—C20A—O4A	2.0 (3)	O3B—C19B—C20B—C21B	−177.6 (2)
O4A—C20A—C21A—C16A	175.55 (17)	C18B—C19B—C20B—O4B	−178.1 (2)
C19A—C20A—C21A—C16A	−5.4 (3)	O3B—C19B—C20B—O4B	2.8 (3)
N2A—C16A—C21A—C20A	−174.61 (18)	O4B—C20B—C21B—C16B	179.54 (19)
C17A—C16A—C21A—C20A	1.6 (3)	C19B—C20B—C21B—C16B	−0.1 (3)
C19A—O3A—C22A—C23A	−40.4 (3)	N2B—C16B—C21B—C20B	178.6 (2)
C20A—O4A—C23A—C22A	−51.6 (2)	C17B—C16B—C21B—C20B	−1.6 (3)
O3A—C22A—C23A—O4A	62.5 (2)	C19B—O3B—C22B—C23B	−26.1 (8)
C4A—C5A—O2B—C15B	−78.6 (2)	O3B—C22B—C23B—O4B	39.4 (13)
C6A—C5A—O2B—C15B	103.76 (19)	C20B—O4B—C23B—C22B	−29.3 (12)
C1B^ii^—C2B—C3B—C4B^ii^	−3.5 (3)	C19B—O3B—C22C—C23C	11.0 (8)
C7B—C2B—C3B—C4B^ii^	173.31 (17)	C20B—O4B—C23C—C22C	39.4 (11)
C1B^ii^—C2B—C3B—O1B	−179.17 (15)	O3B—C22C—C23C—O4B	−34.0 (13)

Symmetry codes: (i) −*x*+1, *y*, −*z*+1/2; (ii) −*x*+1, *y*, −*z*+3/2.

### Hydrogen-bond geometry (Å, º)

C*g*1 is the centroid of the ring C1*B*–C6*B*; C*g*2 is the centroid of the ring C14*A*–C17*A*/N1*A*/N2*A*.

**Table:**

*D*—H···*A*	*D*—H	H···*A*	*D*···*A*	*D*—H···*A*
C21*B*—H21*B*···O3*A*^iii^	0.95	2.50	3.307 (3)	143
C18*A*—H18*A*···O3*A*^iii^	0.95	2.40	3.302 (2)	158
C8*B*^iv^—H8*B*2^iv^···C20*A*	0.99	2.71	3.693 (3)	170
C23*A*—H23*A*···C*g*1^v^	0.99	2.88	3.530 (4)	124
C12*B*^vi^—H12*D*^vi^···O1*A*	0.99	2.71	3.414 (3)	128
C2*S*—H2*S*···C*g*2^i^	0.95	2.67	3.609 (3)	171

Symmetry codes: (i) −*x*+1, *y*, −*z*+1/2; (iii) −*x*+1, −*y*+2, −*z*+1; (iv) *x*+1/2, *y*+1/2, *z*; (v) −*x*+3/2, *y*+1/2, −*z*+3/2; (vi) −*x*+1, −*y*+1, −*z*+1.
